# Supplementary material for: Home-based screening tools for amblyopia: a systematic review protocol
Source: BMJ Open. 2021 Aug 27;11(8):e051830. doi: 10.1136/bmjopen-2021-051830 (PMC8404433; doi:10.1136/bmjopen-2021-051830)
Supplement: Supplementary data [file bmjopen-2021-051830supp001.pdf]

Appendices

Appendix 1: Sample search strategy – MEDLINE

1. exp Amblyopia/ or amblyop*.mp. or amblyog*.mp.

2. ((assess* or diagnos* or detect* or screen* or test*) adj4 (vision* or visual*)).mp.

3. exp visual acuity/

4. exp vision screening/ or screen*.mp.

5. ((assess* or diagnos* or detect*or screen* or test*) adj4 (communit* or population*)).mp.

6. ((assess* or diagnos* or detect* or screen* or test*) adj4 program*).mp.

7. 2 or 3 or 4 or 5 or 6

8. (home* or internet* or web* or app* or computer* or smartphone* or mobile*).mp. or Mobile Applications/ or Smartphone Applications/

9. 1 and 7 and 8

10. limit 9 to "all child (0 to 18 years)"

Sii S, *et al. BMJ Open* 2021; 11:e051830. doi: 10.1136/bmjopen-2021-051830

Appendix 2: Screening questionnaire

Instructions for screeners: Tick the appropriate box per screening question. If "no" at any stage, exclude. If "yes" or "unclear", proceed to next stage; if "yes" at Stage 3, include. If "unclear" at Stage 3, contact study authors for further information and/or seek verdict from third arbitrator.

Stage 1: Title Screening

1a) Does the study represent Level IV evidence or above, i.e. case series, cohort studies, case-control studies, randomised controlled trials (RCTs) and systematic reviews?

Yes	
No	
Unclear	

1b) Does the study involve home-based screening methods for amblyopia in children under 18 years of age?

Yes	
No	
Unclear	

Stage 2: Abstract Screening

2a) Does the study represent Level IV evidence or above, i.e. case series, cohort studies, case-control studies, randomised controlled trials (RCTs) and systematic reviews?

Yes	
No	
Unclear	

2b) Does the study involve home-based screening methods for amblyopia in children under 18 years of age?

Yes	
No	
Unclear	

Stage 3: Full text Screening

3a) Does the study represent Level IV evidence or above, i.e. case series, cohort studies, case-control studies, randomised controlled trials (RCTs) and systematic reviews?

Yes	
No	
Unclear	

3b) Does the study involve home-based screening methods for amblyopia in children under 18 years of age?

Yes	
No	
Unclear	

Appendix 3: Data extraction tool, adapted from the Cochrane Collaboration

Methods

Aim of study	
Study design	
Inclusion criteria	
Exclusion criteria	
Methods of recruitment	
Methods of randomisation (if applicable)	
Number of patients	

Specific to diagnostic accuracy studies

Personnel conducting index test	
Personnel conducting gold standard test	
Subjects receiving test	
Blinding (if applicable)	
Index test	
Reference test	
Personnel interpreting test results	
Withdrawal rate/loss to follow up<br>If not specified state so	
Sensitivity(including CI)	
Specificity(including CI)	
False positive	
False negative	
Correlation coefficient including p-values	

Specific to other evaluation studies

Personnel conducting index test	
Personnel conducting reference test	
Subjects receiving test	
Blinding (if applicable)	
Index test	
Reference test	
Personnel interpreting test results	
Withdrawal rate/loss to follow up	
Results reported (appropriate statistical measures)	
Economic consideration/cost required	

Sii S, *et al. BMJ Open* 2021; 11:e051830. doi: 10.1136/bmjopen-2021-051830

Appendix 4: QUADAS-2 tool

Domain 1: Patient selection

A. Risk of bias<br>Describe methods of patient selection:	Low	High	Unclear
Was a consecutive or random sample of patients enrolled?			
Was a case-control design avoided?			
Did the study avoid inappropriate exclusions?			
Could the selection of patients have introduced bias?			
B. Concerns regarding applicability	Low	High	Unclear
Is there concern that the included patients do not represent the actual spectrum of patients in practice?			

Domain 2: Index test(s)

Risk of bias<br>Describe the index text and how is it conducted and interpreted:	Low	High	Unclear
Were the index test results interpreted without knowledge of the results of the reference standard?			
If a threshold was used, was it pre-specified?			
Was the index test described in sufficient detail to enable replication of the test?			
Could the index test, its conduct, or its interpretation have introduced bias?			
B. Concerns regarding applicability	Low	High	Unclear
Is there concern that the index test, its conduct, or interpretation differ from the review question?			

Domain 3: Reference standard

Risk of bias<br>Describe the index text and how is it conducted and interpreted:	Low	High	Unclear
Is the reference standard likely to correctly classify the target condition?			
Were the reference standard results interpreted without knowledge of the results of the index test?			
Was the standard test described in sufficient detail to enable replication of the test?			
Could the reference standard, its conduct, or its interpretation have introduced bias?			
B. Concerns regarding applicability	Low	High	Unclear
Is there concern that the target condition as defined by the reference standard does not match the review question?			

Domain 4: Flow and timing

Risk of bias	Low	High	Unclear
Was there an appropriate interval between index test(s) and reference standard, so that the target condition did not change between two tests?			
Did all patients receive a reference standard?			
Did all patients receive the same reference standard?			
Were all patients included in the analysis?			
Could the patient flow have introduced bias?			

Sii S, *et al. BMJ Open* 2021; 11:e051830. doi: 10.1136/bmjopen-2021-051830
