# Supplementary material for: Home-based screening tools for amblyopia: a systematic review protocol
Source: BMJ Open. 2021 Aug 27;11(8):e051830. doi: 10.1136/bmjopen-2021-051830 (PMC8404433; doi:10.1136/bmjopen-2021-051830)
Supplement: Author's manuscript [file bmjopen-2021-051830.draft_revisions.pdf]

BMJ Open is committed to open peer review. As part of this commitment we make the peer review history of every article we publish publicly available.

When an article is published we post the peer reviewers' comments and the authors' responses online. We also post the versions of the paper that were used during peer review. These are the versions that the peer review comments apply to.

The versions of the paper that follow are the versions that were submitted during the peer review process. They are not the versions of record or the final published versions. They should not be cited or distributed as the published version of this manuscript.

BMJ Open is an open access journal and the full, final, typeset and author-corrected version of record of the manuscript is available on our site with no access controls, subscription charges or pay-per-view fees (http://bmjopen.bmj.com).

If you have any questions on BMJ Open's open peer review process please email info.bmjopen@bmj.com

BMJ Open

Home-based screening tools for amblyopia: a systematic review protocol

Journal:	*BMJ Open*
Manuscript ID	bmjopen-2021-051830
Article Type:	Protocol
Date Submitted by the Author:	29-Mar-2021
Complete List of Authors:	Sii, Samantha; Kettering General Hospital NHS Trust, ophthalmology<br>Chean, Chung Shen; Northampton General Hospital, Department of Ophthalmology<br>Kuht, Helen; Leicester Royal Infirmary<br>Thomas, Mervyn; Leicester Royal Infirmary<br>Rufai, Sohaib; Leicester Royal Infirmary, Clinical and Academic Department of Ophthalmology; Great Ormond Street Hospital for Children, Clinical and Academic Department of Ophthalmology
Keywords:	Community child health < PAEDIATRICS, Paediatric ophthalmology < OPHTHALMOLOGY, Information technology < BIOTECHNOLOGY & BIOINFORMATICS, Telemedicine < BIOTECHNOLOGY & BIOINFORMATICS

SCHOLARONE™
Manuscripts

BMJ

I, the Submitting Author has the right to grant and does grant on behalf of all authors of the Work (as defined in the below author licence), an exclusive licence and/or a non-exclusive licence for contributions from authors who are: i) UK Crown employees; ii) where BMJ has agreed a CC-BY licence shall apply, and/or iii) in accordance with the terms applicable for US Federal Government officers or employees acting as part of their official duties; on a worldwide, perpetual, irrevocable, royalty-free basis to BMJ Publishing Group Ltd ("BMJ") its licensees and where the relevant Journal is co-owned by BMJ to the co-owners of the Journal, to publish the Work in this journal and any other BMJ products and to exploit all rights, as set out in our licence.

The Submitting Author accepts and understands that any supply made under these terms is made by BMJ to the Submitting Author unless you are acting as an employee on behalf of your employer or a postgraduate student of an affiliated institution which is paying any applicable article publishing charge ("APC") for Open Access articles. Where the Submitting Author wishes to make the Work available on an Open Access basis (and intends to pay the relevant APC), the terms of reuse of such Open Access shall be governed by a Creative Commons licence – details of these licences and which Creative Commons licence will apply to this Work are set out in our licence referred to above.

Other than as permitted in any relevant BMJ Author's Self Archiving Policies, I confirm this Work has not been accepted for publication elsewhere, is not being considered for publication elsewhere and does not duplicate material already published. I confirm all authors consent to publication of this Work and authorise the granting of this licence.

For peer review only

Title: Home-based screening tools for amblyopia: a systematic review protocol

Authors:

Samantha Sii[1], Chung Shen Chean[2], Helen Kuht[3], Mervyn G Thomas[3*], Sohaib R Rufai[3,4*]

[1]Department of Ophthalmology, Kettering General Hospital, Kettering, United Kingdom.

[2]Department of Ophthalmology, Northampton General Hospital, Cliftonville, Northampton, United Kingdom.

[3] University of Leicester, Ulverscroft Eye Unit, Leicester Royal Infirmary, Leicester, United Kingdom.

[4]Clinical and Academic Department of Ophthalmology, Great Ormond Street Hospital for Children, London, United Kingdom.

*Co-Senior Author

Correspondence: Dr Mervyn G Thomas; mt350@leicester.ac.uk; Dr Sohaib R Rufai; Sohaib.Rufai@nhs.net.

Type of manuscript: Protocol

For peer review only

ABSTRACT

Introduction:

Amblyopia is an important public health issue associated with vision loss and detrimental impact on the physical and mental wellbeing of children. The gold standard for diagnosis of amblyogenic conditions involves screening by ophthalmologists and orthoptists. Recent advances in technology have enabled the use of home-based screening tools to detect these conditions at an early stage. Here, we propose a systematic review aiming to evaluate the accuracy and reliability of home-based screening tools comparing to the existing gold standard.

Methods & Analysis:

We aim to search for studies involving amblyopia home-based screening tools used in children under 18 years of age. Oxford Centre for Evidence-Based Medicine Level 4 evidence and above will be included. The following platforms will be searched from inception to 31st May 2021 – the proposed search date: Medline, The Cochrane Library, Embase, Web of Science Core Collection and Clinicaltrials.gov. Two independent reviewers will identify studies for inclusion. The screening will be performed from 31st of May 2021 to 1st of July 2021. We aim to complete our data analysis by the 30th of September 2021. Risk of bias will be assessed using the QUADAS-2 tool for diagnostic accuracy studies. Our primary outcome measure is the diagnostic accuracy of amblyopia home-based screening tools, whilst secondary outcome measures include validity, feasibility, reproducibility, and cost effectiveness.

Ethics & Dissemination:

Ethical approval is not required as no primary data will be collected. The findings will be disseminated through presentations at scientific meetings and peer-reviewed journal publication.

Prospero registration number: CRD42021233511

For peer review only

For peer review only - http://bmjopen.bmj.com/site/about/guidelines.xhtml

Article Summary:

Strengths and limitations of this study

- This will be the first systematic review evaluating the accuracy and reliability of home-based screening tools for amblyopia.
- Published and unpublished literature without language or time restrictions will be included.
- Our methodology is based on principles extracted from the Cochrane Collaboration.
- The main limitation could be a scarcity of randomised controlled trials and diagnostic accuracy studies involving home-based screening tools.
- The broad search strategy should help ensure that all relevant literature is included.

For peer review only

For peer review only - http://bmjopen.bmj.com/site/about/guidelines.xhtml

INTRODUCTION

Amblyopia is one of the commonest preventable causes of vision loss affecting children. It continues to represent a significant public health issue, affecting 2-5% of the population.[1,2,3]Amblyopia is usually associated with visual deprivation early in life[4] due to amblyogenic risk factors, which include uncorrected refractive errors, astigmatism, congenital pathologies or media opacities that causes stimulus deprivation, and abnormal binocular interaction from strabismus.[5,6,7] Children with amblyopia are characterised by monocular or binocular visual deficits, including reduced visual acuity, contrast sensitivity, contour integration and depth perception without observable ocular pathologic features.[8]

Amblyopia is largely asymptomatic initially, but untreated amblyopia resulting in vision loss can lead to problems at school, reduced quality of life, lifelong consequences on future occupation choices and mental health issues.[9,10] Contrary to the traditional notion that amblyopia treatment may be ineffective for children above 7 years old,[11] the Paediatric Eye Disease Investigator Group (PEDIG) studies showed that treatment of amblyopia may still be effective in children aged 7 to 17 years,[12,13] with the effectiveness of treatment becoming significantly reduced with time.[14]

Screening for amblyopia was introduced in the 1950s and advocated in many countries.[15] Many screening programmes have been unsuccessful, with an estimation of less than 25% of preschool-aged children being screened through a government or private program in the United States.[16] In addition, up to 60% of primary care providers do not perform vision screening on preschool-aged children, and others perform screening inconsistently.[16] Significant barriers to traditional vision screening include cost, limited access to healthcare and a limited number of qualified screeners available.[17] Hence, a variety of methodologies for vision screening have been trialled, including the use of home-based amblyopia screening tools, to help overcome these barriers to vision screening.[18]

The coronavirus disease 2019 (COVID-19) pandemic illustrates the increasingly important role of telemedicine as a method of clinician-patient interaction. The use of home-based screening tools for amblyopia are increasingly advocated as social distancing is practised to minimise the risk of viral transmission.[19,20] Furthermore, COVID-19 related restrictions and lockdowns may have resulted in many children missing opportunities for amblyopia screening.[19] Home-based screening may represent an effective solution,[21] but its role has not been rigorously assessed

For peer review only - http://bmjopen.bmj.com/site/about/guidelines.xhtml

by systematic review. Here, we propose a systematic review to evaluate the diagnostic accuracy and reliability of home-based amblyopia screening tools.

METHODS AND ANALYSIS

This protocol is drafted according to the Preferred Reporting Items for Systematic Review and Meta-Analysis Protocols (PRISMA-P) checklist.[22]

Eligibility criteria for studies

The eligibility criteria for this systematic review are defined according to the Population, Intervention, Comparison, Outcome and Study Design (PICOS)[23] strategy, as outlined in **Table 1**.

Table 1 Eligibility criteria of studies

PICOS strategy	Inclusion criteria	Exclusion criteria
**Population**	Studies involving screening for amblyopia in children aged under 18 years old.	Studies involving adults aged 18 years old and above.
**Intervention**	Home-based screening tools including:<br>i) Internet or web-based visual acuity screening tools;<br>ii) Mobile applications used to screen for conditions contributing to amblyopia;<br>iii) Novel home-based gadgets or instruments used to screen for conditions contributing to amblyopia.	Orthoptist-led or ophthalmologist-led amblyopia screening tests including:<br>i) Standard logMAR (or equivalent) visual acuity measurement charts;<br>ii) Comprehensive eye examination using slit lamp or ocular motility examination;<br>iii) Autorefractors or photoscreeners.

For peer review only - http://bmjopen.bmj.com/site/about/guidelines.xhtml

**Comparison/Control**	Orthoptist-led or ophthalmologist-led amblyopia screening.	Not applicable.
**Outcomes**	Primary outcome measure: Diagnostic accuracy of home-based amblyopia screening tools.<br><br>Secondary outcome measures, where available: validity, feasibility, reproducibility, cost effectiveness.	i) Studies not reporting outcomes related to amblyopia screening;<br>ii) Epidemiological studies of amblyopia.
**Study Design**	According to the Oxford Centre for Evidence-Based Medicine (CEBM) Level 4 evidence and above will be included.[24]	CEBM Level 5 evidence and below will be excluded.

Information sources

The following electronic searches will be included in this systematic review:

I. Ovid MEDLINE (1946 to present)
II. The Cochrane Library
III. Embase (1974 to present)
IV. Web of Science Core Collection (1970 to present).
V. Clinicaltrials.gov

Other sources

We will include full-text articles without time and language restrictions. We will exclude conference abstracts, opinion pieces, guidelines and editorials.

To ensure literature saturation, references of included studies will be searched and included if eligible. Authors of published studies with insufficient data will be contacted to attempt to

For peer review only - http://bmjopen.bmj.com/site/about/guidelines.xhtml

obtain relevant outcome data. If there is no response from these authors after 14 days, there will be a second attempt to establish contact. If there is still no response after 14 days, these studies will be excluded.

Search strategy

The search strategy was formulated in consultation with a research services consultant with experience in systematic review. The search terms 'amblyopia', 'vision screening', 'home', 'web', 'internet' 'app', 'smartphone', and 'mobile' were entered into the electronic search platforms. A full sample search strategy is available in the online supplementary **Appendix 1**.

Study records

Data Management

EndNote V.X9 (Thomson Reuters, New York, New York, USA) reference management software will be used for data management.

Selection of studies

Two independent screeners (SS and CSC) shall follow a three-stage screening method, according to a screening questionnaire (**Appendix 2**). After title and abstract screening, SS and CSC will compare and attempt to resolve any disagreements on the inclusion of articles, where applicable. If any disagreement remains, opinion will be sought from the third arbitrator (HJK). We aim to execute the search on 31st May 2021 and complete the screening process by 1st July 2021.

Data collection

Our data collection tool adapted from the Cochrane Collaboration is included in online supplementary **Appendix 3**. A preliminary data collection form was first drawn and piloted among the authors of this study before use. The following data will be collected: study design, number of included patients, duration of study, method of intervention used, index test and reference test where applicable. Outcomes pertinent to the quality of diagnostic studies

For peer review only - http://bmjopen.bmj.com/site/about/guidelines.xhtml

including investigators conducting the test, subjects receiving the test, method of interpretation, blinding of participants or investigators and withdrawal rate will also be included.

Outcome Measures and Prioritisation

Our primary outcome measure of interest will be the diagnostic accuracy of home-based screening tools in detecting amblyopia as compared to the existing gold standard, which is orthoptist-led or ophthalmologist-led amblyopia screening. Outcomes from diagnostic accuracy studies such as sensitivity, specificity, positive predictive value (PPV), and negative predictive value (NPV) will be prioritised as the primary outcome as they will translate into meaningful endpoints for comparing the effectiveness of home-based screening tools against the gold standard. The secondary outcome measures may include validity, feasibility, reproducibility, and cost effectiveness of these home-based screening tools. These will be reported in appropriate statistical measures if represented by studies with large enough sample sizes. As some outcomes may be reported as a composite measure, we will extract all composites and individual outcomes as reported in the included studies.

Risk of bias assessment

Risk of bias assessment will be performed for diagnostic accuracy studies only. The quality of diagnostic accuracy studies will be assessed using the QUADAS-2 tool (**Appendix 4**).[25] These judgments will be made independently by two review authors (SS, CSC) and any disagreements resolved by the third arbitrator (HJK). If our risk of bias assessment shows lack of good quality studies with adequate sample sizes, statistical measures will not be summarised quantitatively and vice versa.

Data analysis

Scoping searches suggest that mainly observational studies will be returned by our search strategy with few relevant randomised controlled trials (RCTs). Weighted means for primary outcome measures (such as sensitivity, specificity, PPV, NPV) will only be calculated if multiple RCTs or good quality large scale prospective studies are identified. Otherwise, we shall perform a qualitative review summarising the available evidence of studies , including

For peer review only - http://bmjopen.bmj.com/site/about/guidelines.xhtml

tables summarising the characteristics and results of included studies as well as relevant p-values. This will be followed by a narrative synthesis of secondary outcome measures such as validity, feasibility, reproducibility, or cost effectiveness of home-based amblyopia screening tools. We aim to complete our data analysis by 30th September 2021.

Confidence in cumulative estimate

The quality of evidence for all outcomes will be judged using the Grading of Recommendations Assessment, Development and Evaluation working group methodology (GRADE).[26] This will be judged as high (further research is very unlikely to change our confidence in the estimate of effect), moderate (further research is likely to have an important impact on our confidence in the estimate of effect and may change the estimate), low (further research is very likely to have an important impact on our confidence in the estimate of effect and is likely to change the estimate), or very low (very uncertain about the estimate of effect).

Patient and Public Involvement statement

As this systematic review does not involve recruitment of patients for research, patient and public involvement has not been arranged.

Ethics and dissemination

As this systematic review does not involve recruiting patients, independent ethical approval is not required. The findings of this systematic review shall be disseminated through presentations at scientific meetings, as well as peer-reviewed journal publication. Any data generated from this systematic review can be made available from the corresponding author on reasonable request.

Discussion

To our knowledge, this is the first systematic review aiming to compare home-based screening tools and existing screening services offered through ophthalmologists and orthoptists to diagnose amblyopia. We adhered to the PRISMA-P checklist in drafting this protocol. Through publication of this protocol, we aim to provide transparency in the methodology of our systematic review. This should increase internal validity by preventing publication bias and help avoid study duplication.

Word count: 1990 words

References

1. Powell C, Hatt SR. Vision screening for amblyopia in childhood. *Cochrane Database Syst Rev* 2009; 8:(3). https://www.cochranelibrary.com/cdsr/doi/10.1002/14651858.CD005020.pub3/full (accessed on 22 Feb 2021)
2. Friedman DA, Repka MX, Katz J, et al: Prevalence of amblyopia and strabismus in White and African-American children aged 6 through 71 months: The Baltimore Pediatric Eye Disease Study. *Ophthalmology* 2009; 116: 2128-2134
3. Multi-ethnic Pediatric Eye Disease Study Group. Prevalence of amblyopia and strabismus in African American and Hispanic children ages 6 to 72 months the multi-ethnic pediatric eye disease study. *Ophthalmology* 2008; 115: 1229-1236
4. Levi DM. Progress and paradigm shifts in spatial vision over the 20 years of ECVP. *Perception* 1999;28(12):1443-59.
5. Rahi J, Logan S, Timms C et al. Risk, causes, and outcomes of visual impairment after loss of vision in the non-amblyopic eye: a population-based study. *Lancet* 2002; 360 (9333):597-602.
6. Repka M, Simons K, and Kraker R. Laterality of amblyopia. *Am J Ophthalmol* 2010; 150: pp. 270-274
7. Shaw DE, Fielder AR, Minshull C et al. Amblyopia – Factors influencing age of presentation. *Lancet* 1988; 2: 207-209
8. Lan W, Zhao F, Li Z et al. Validation and cost-effectiveness of a home-based screening system for amblyopia. *Ophthalmology* 2012 ;119(6):1265-71.
9. Jonas DE, Amick HR, Wallace IF et al. Vision screening in children aged 6 months to 5 years: evidence report and systematic review for the US Preventive Services Task Force. *JAMA* 2017; 318(9):845-58.
10. Tandon AK, Velez FG, Isenberg SJ, et al: Binocular inhibition in strabismic patients is associated with diminished quality of life. *J AAPOS* 2014; 18:423-426
11. Assaf AA. The sensitive period: transfer of fixation after occlusion for strabismic amblyopia. *Br J Ophthalmol* 1982; 66: 64–70.
12. Scheiman MM, Hertle RW, Beck RW et al. Randomized trial of treatment of amblyopia in children aged 7 to 17 years. *Arch Ophthalmol* 2005 Apr 1;123(4):437-47.
13. Scheiman MM, Hertle RW, Kraker RT, et al. Patching vs atropine to treat amblyopia in children aged 7 to 12 years: a randomized trial. *Arch Ophthalmol* 2008;126(12):1634-1642.

For peer review only - http://bmjopen.bmj.com/site/about/guidelines.xhtml

14. Holmes JM, Lazar EL, Melia BM, et al. Effect of age on response to amblyopia treatment in children. *Arch Ophthalmol* 2011;129(11):1451-57
15. Gundersen T. Glaucoma and amblyopia ex anopsia, two preventable forms of blindness. *J Am Med Assoc* 1954; 156: 933–5.
16. Hered RW, Murphy S, Clancy M. Comparison of the HOTV and Lea symbols charts for preschool vision screening. *J Pediatr Ophthalmol Strabismus* 1997; 34: 24-8.
17. Longmuir SQ, Pfeifer W, Shah SS et al. Validity of a layperson-administered Web-based vision screening test. *J AAPOS* 2015 ; 19(1): 29-32.
18. Tamez-Tamez VE, Ruiz-Lozano RE. Evaluating amblyopia during the era of COVID-19. *Graefes Arch Clin Exp Ophthalmol*. 2020;258(12):2857-2859.
19. Painter, S, Ramm, L, Wadlow, L. et al Parental Home Vision Testing of Children During Covid-19 Pandemic. *British and Irish Orthoptic Journal*, 17(1), 13–19
20. Samanta A, Mauntana S, Barsi Z, Yarlagadda B, Nelson PC. Is your vision blurry? A systematic review of home-based visual acuity for telemedicine. *J Telemed Telecare* 2020 Jan 1:1357633X20970398.
21. Kawamoto, K, Stanojcic, N, Li, JO et al. Visual Acuity Apps for Rapid Integration in Teleconsultation Services in all Resource Settings, *Asia Pac J Ophthalmol*:2021 https://journals.lww.com/apjoo/pages/articleviewer.aspx?year=9000&issue=00000&article=99702&type=Abstract (accessed on 29 Feb 2021)
22. Moher D, Shamseer L, Clarke M, Ghersi D, Liberati A, Petticrew M, Shekelle P, Stewart LA. Preferred Reporting Items for Systematic Review and Meta-Analysis Protocols (PRISMA-P) 2015 statement. *Syst Rev.* 2015;4(1):1
23. Higgins JP, Green S. Defining the review question and developing criteria for including studies, in Cochrane handbook for systematic reviews of interventions. *Wiley Online Library*, 2008: 1–83.
24. CEBM. Oxford Centre for Evidence-based Medicine – Levels of Evidence (March 2009). taken from https://www. cebm. net/ 2009/ 06/oxford- centre- evidence- based-medicine- levels- evidence- march-2009/ (accessed 29 Feb 2021)
25. Whiting P, Rutjes A, Westwood M, et al. QUADAS-2: an updated quality assessment tool for diagnostic accuracy studies. In: *Abstracts of the 19th Cochrane Colloquium*; 2011 19-22 Oct. https://abstracts.cochrane.org/2011-madrid/quadas-2-updated-quality-assessment-tool-diagnostic-accuracy-studies (accessed on 27 Feb 2011)

For peer review only - http://bmjopen.bmj.com/site/about/guidelines.xhtml

26. Iorio A, Spencer FA, Falavigna M, *et al*. Use of grade for assessment of evidence about prognosis: rating confidence in estimates of event rates in broad categories of patients. *BMJ* 2015; 350: h870.

For peer review only - http://bmjopen.bmj.com/site/about/guidelines.xhtml

Author Contributions:

SS: Concept, methodology, protocol writing and final approval.

CSC: Protocol writing, critical revision and final approval.

HJK: Critical revision and final approval.

MGT: Supervision, methodology, critical revision, final approval.

SRR: Supervision, concept, methodology, critical revision, final approval.

Funding Statement:

SRR is funded by a National Institute for Health Research (NIHR) Doctoral Fellowship Award (NIHR300155). MGT is supported by the NIHR (CL-2017-11-003) and the Ulverscroft foundation. This paper presents independent research funded by the National Institute for Health Research (NIHR). The views expressed are those of the author(s) and not necessarily those of, the Ulverscroft Foundation, the NHS, the NIHR or the Department of Health and Social Care.

Conflict of Interest: None declared

Data Statement:

As this is a protocol manuscript of a planned systematic review, there are no data available.

Acknowledgement:

We thank Selina Lock, Research Services Consultant at the University of Leicester David Wilson Library, for providing guidance in our systematic search strategy.

For peer review only - http://bmjopen.bmj.com/site/about/guidelines.xhtml

Appendices

Appendix 1: Sample search strategy – MEDLINE

1. exp Amblyopia/ or amblyop*.mp. or amblyog*.mp.

2. ((assess* or diagnos* or screen* or test*) adj4 (vision* or visual*)).mp.

3. exp vision test/

4. exp vision screening/ or screen*.mp.

5. ((assess* or diagnos* or screen* or test*) adj4 (communit* or population*)).mp.

6. ((assess* or diagnos* or screen* or test*) adj4 program*).mp.

7. 2 or 3 or 4 or 5 or 6

8. (home* or internet* or web* or app* or computer* or smartphone* or mobile*).mp. or Mobile Applications/ or Smartphone Applications/

9. 1 and 7 and 8

10. limit 9 to "all child (0 to 18 years)"

For peer review only

For peer review only - http://bmjopen.bmj.com/site/about/guidelines.xhtml

Appendix 2: Screening questionnaire

Instructions for screeners: Tick the appropriate box per screening question. If "no" at any stage, exclude. If "yes" or "unclear", proceed to next stage; if "yes" at Stage 3, include. If "unclear" at Stage 3, contact study authors for further information and/or seek verdict from third arbitrator.

Stage 1: Title Screening

1a) Does the study represent Level IV evidence or above, i.e. case series, cohort studies, case-control studies, randomised controlled trials (RCTs) and systematic reviews?

Yes	
No	
Unclear	

1b) Does the study involve home-based screening methods for amblyopia in children under 18 years of age?

Yes	
No	
Unclear	

Stage 2: Abstract Screening

2a) Does the study represent Level IV evidence or above, i.e. case series, cohort studies, case-control studies, randomised controlled trials (RCTs) and systematic reviews?

Yes	
No	
Unclear	

2b) Does the study involve home-based screening methods for amblyopia in children under 18 years of age?

Yes	
No	
Unclear	

Stage 3: Full text Screening

3a) Does the study represent Level IV evidence or above, i.e. case series, cohort studies, case-control studies, randomised controlled trials (RCTs) and systematic reviews?

Yes	
No	
Unclear	

3b) Does the study involve home-based screening methods for amblyopia in children under 18 years of age?

Yes	
No	
Unclear	

For peer review only
For peer review only - http://bmjopen.bmj.com/site/about/guidelines.xhtml

Appendix 3: Data extraction tool, adapted from the Cochrane Collaboration

Methods

Aim of study	
Study design	
Inclusion criteria	
Exclusion criteria	
Methods of recruitment	
Methods of randomisation (if applicable)	
Number of patients	

Specific to diagnostic accuracy studies

Personnel conducting index test	
Personnel conducting gold standard test	
Subjects receiving test	
Blinding (if applicable)	
Index test	
Reference test	
Personnel interpreting test results	
Withdrawal rate/loss to follow up<br>If not specified state so	
Sensitivity(including CI)	
Specificity(including CI)	
False positive	
False negative	
Correlation coefficient including p-values	

Specific to other evaluation studies

Personnel conducting index test	
Personnel conducting reference test	
Subjects receiving test	
Blinding (if applicable)	
Index test	
Reference test	
Personnel interpreting test results	
Withdrawal rate/loss to follow up	
Results reported (appropriate statistical measures)	
Economic consideration/cost required	

For peer review only

For peer review only - http://bmjopen.bmj.com/site/about/guidelines.xhtml

Appendix 4: QUADAS-2 tool

Domain 1: Patient selection

A. Risk of bias<br>Describe methods of patient selection:	Low	High	Unclear
Was a consecutive or random sample of patients enrolled?			
Was a case-control design avoided?			
Did the study avoid inappropriate exclusions?			
Could the selection of patients have introduced bias?			
B. Concerns regarding applicability	Low	High	Unclear
Is there concern that the included patients do not represent the actual spectrum of patients in practice?			

Domain 2: Index test(s)

Risk of bias<br>Describe the index text and how is it conducted and interpreted:	Low	High	Unclear
Were the index test results interpreted without knowledge of the results of the reference standard?			
If a threshold was used, was it pre-specified?			
Was the index test described in sufficient detail to enable replication of the test?			
Could the index test, its conduct, or its interpretation have introduced bias?			
B. Concerns regarding applicability	Low	High	Unclear
Is there concern that the index test, its conduct, or interpretation differ from the review question?			

For peer review only

Domain 3: Reference standard

Risk of bias<br>Describe the index text and how is it conducted and interpreted:	Low	High	Unclear
Is the reference standard likely to correctly classify the target condition?			
Were the reference standard results interpreted without knowledge of the results of the index test?			
Was the standard test described in sufficient detail to enable replication of the test?			
Could the reference standard, its conduct, or its interpretation have introduced bias?			
B. Concerns regarding applicability	Low	High	Unclear
Is there concern that the target condition as defined by the reference standard does not match the review question?			

Domain 4: Flow and timing

Risk of bias	Low	High	Unclear
Was there an appropriate interval between index test(s) and reference standard, so that the target condition did not change between two tests?			
Did all patients receive a reference standard?			
Did all patients receive the same reference standard?			
Were all patients included in the analysis?			
Could the patient flow have introduced bias?			

For peer review only

For peer review only - http://bmjopen.bmj.com/site/about/guidelines.xhtml

For peer review only

For peer review only - http://bmjopen.bmj.com/site/about/guidelines.xhtml

Reporting checklist for protocol of a systematic review and meta analysis.

Based on the PRISMA-P guidelines.

Instructions to authors

Complete this checklist by entering the page numbers from your manuscript where readers will find each of the items listed below.

Your article may not currently address all the items on the checklist. Please modify your text to include the missing information. If you are certain that an item does not apply, please write "n/a" and provide a short explanation.

Upload your completed checklist as an extra file when you submit to a journal.

In your methods section, say that you used the PRISMA-Preporting guidelines, and cite them as:

Moher D, Shamseer L, Clarke M, Ghersi D, Liberati A, Petticrew M, Shekelle P, Stewart LA. Preferred Reporting Items for Systematic Review and Meta-Analysis Protocols (PRISMA-P) 2015 statement. Syst Rev. 2015;4(1):1.

		Reporting Item	Page Number
**Title**			
Identification	#1a	Identify the report as a protocol of a systematic review	1
Update	#1b	If the protocol is for an update of a previous systematic review, identify as such	NA

For peer review only

**Registration**			
	#2	If registered, provide the name of the registry (such as PROSPERO) and registration number	2
**Authors**			
Contact	#3a	Provide name, institutional affiliation, e-mail address of all protocol authors; provide physical mailing address of corresponding author	1
Contribution	#3b	Describe contributions of protocol authors and identify the guarantor of the review	13
**Amendments**			
	#4	If the protocol represents an amendment of a previously completed or published protocol, identify as such and list changes; otherwise, state plan for documenting important protocol amendments	NA
**Support**			
Sources	#5a	Indicate sources of financial or other support for the review	13
Sponsor	#5b	Provide name for the review funder and / or sponsor	13
Role of sponsor or funder	#5c	Describe roles of funder(s), sponsor(s), and / or institution(s), if any, in developing the protocol	13
**Introduction**			
Rationale	#6	Describe the rationale for the review in the context of what is	4

For peer review only

		already known	
Objectives	#7	Provide an explicit statement of the question(s) the review will address with reference to participants, interventions, comparators, and outcomes (PICO)	5-6
**Methods**			
Eligibility criteria	#8	Specify the study characteristics (such as PICO, study design, setting, time frame) and report characteristics (such as years considered, language, publication status) to be used as criteria for eligibility for the review	5-6
Information sources	#9	Describe all intended information sources (such as electronic databases, contact with study authors, trial registers or other grey literature sources) with planned dates of coverage	6
Search strategy	#10	Present draft of search strategy to be used for at least one electronic database, including planned limits, such that it could be repeated	7
Study records - data management	#11a	Describe the mechanism(s) that will be used to manage records and data throughout the review	7
Study records - selection process	#11b	State the process that will be used for selecting studies (such as two independent reviewers) through each phase of the review (that is, screening, eligibility and inclusion in meta-analysis)	7
Study records - data collection	#11c	Describe planned method of extracting data from reports (such as piloting forms, done independently, in duplicate), any	7

For peer review only

process		processes for obtaining and confirming data from investigators	
Data items	#12	List and define all variables for which data will be sought (such as PICO items, funding sources), any pre-planned data assumptions and simplifications	7
Outcomes and prioritization	#13	List and define all outcomes for which data will be sought, including prioritization of main and additional outcomes, with rationale	8
Risk of bias in individual studies	#14	Describe anticipated methods for assessing risk of bias of individual studies, including whether this will be done at the outcome or study level, or both; state how this information will be used in data synthesis	8
Data synthesis	#15a	Describe criteria under which study data will be quantitatively synthesised	8
Data synthesis	#15b	If data are appropriate for quantitative synthesis, describe planned summary measures, methods of handling data and methods of combining data from studies, including any planned exploration of consistency (such as I2, Kendall's τ)	8
Data synthesis	#15c	Describe any proposed additional analyses (such as sensitivity or subgroup analyses, meta-regression)	8
Data synthesis	#15d	If quantitative synthesis is not appropriate, describe the type of summary planned	8
Meta-bias(es)	#16	Specify any planned assessment of meta-bias(es) (such as publication bias across studies, selective reporting within	NA

For peer review only

		studies)	
Confidence in cumulative evidence	#17	Describe how the strength of the body of evidence will be assessed (such as GRADE)	9

The PRISMA-P elaboration and explanation paper is distributed under the terms of the Creative Commons Attribution License CC-BY. This checklist was completed on 27. March 2021 using https://www.goodreports.org/, a tool made by the EQUATOR Network in collaboration with Penelope.ai

For peer review only

BMJ Open

Home-based screening tools for amblyopia: a systematic review protocol

Journal:	*BMJ Open*
Manuscript ID	bmjopen-2021-051830.R1
Article Type:	Protocol
Date Submitted by the Author:	29-Jul-2021
Complete List of Authors:	Sii, Samantha; Kettering General Hospital NHS Trust, ophthalmology<br>Chean, Chung Shen; Northampton General Hospital, Department of Ophthalmology<br>Kuht, Helen; Leicester Royal Infirmary<br>Thomas, Mervyn; Leicester Royal Infirmary<br>Rufai, Sohaib; Leicester Royal Infirmary, Clinical and Academic Department of Ophthalmology; Great Ormond Street Hospital for Children, Clinical and Academic Department of Ophthalmology
**Primary Subject Heading**:	Ophthalmology
Secondary Subject Heading:	Paediatrics, Health policy, Evidence based practice
Keywords:	Community child health < PAEDIATRICS, Paediatric ophthalmology < OPHTHALMOLOGY, Information technology < BIOTECHNOLOGY & BIOINFORMATICS, Telemedicine < BIOTECHNOLOGY & BIOINFORMATICS

SCHOLARONE™
Manuscripts

I, the Submitting Author has the right to grant and does grant on behalf of all authors of the Work (as defined in the below author licence), an exclusive licence and/or a non-exclusive licence for contributions from authors who are: i) UK Crown employees; ii) where BMJ has agreed a CC-BY licence shall apply, and/or iii) in accordance with the terms applicable for US Federal Government officers or employees acting as part of their official duties; on a worldwide, perpetual, irrevocable, royalty-free basis to BMJ Publishing Group Ltd ("BMJ") its licensees and where the relevant Journal is co-owned by BMJ to the co-owners of the Journal, to publish the Work in this journal and any other BMJ products and to exploit all rights, as set out in our licence.

The Submitting Author accepts and understands that any supply made under these terms is made by BMJ to the Submitting Author unless you are acting as an employee on behalf of your employer or a postgraduate student of an affiliated institution which is paying any applicable article publishing charge ("APC") for Open Access articles. Where the Submitting Author wishes to make the Work available on an Open Access basis (and intends to pay the relevant APC), the terms of reuse of such Open Access shall be governed by a Creative Commons licence – details of these licences and which Creative Commons licence will apply to this Work are set out in our licence referred to above.

Other than as permitted in any relevant BMJ Author's Self Archiving Policies, I confirm this Work has not been accepted for publication elsewhere, is not being considered for publication elsewhere and does not duplicate material already published. I confirm all authors consent to publication of this Work and authorise the granting of this licence.

For peer review only

Title: Home-based screening tools for amblyopia: a systematic review protocol

Authors:

Samantha Sii[1], Chung Shen Chean[2], Helen Kuht[3], Mervyn G Thomas[3*], Sohaib R Rufai[3,4*]

[1]Department of Ophthalmology, Kettering General Hospital, Kettering, United Kingdom.

[2]Department of Ophthalmology, Northampton General Hospital, Cliftonville, Northampton, United Kingdom.

[3] University of Leicester, Ulverscroft Eye Unit, Leicester Royal Infirmary, Leicester, United Kingdom.

[4]Clinical and Academic Department of Ophthalmology, Great Ormond Street Hospital for Children, London, United Kingdom.

*Joint Senior Author

Correspondence: Dr Mervyn G Thomas; mt350@leicester.ac.uk; Dr Sohaib R Rufai; Sohaib.Rufai@nhs.net.

Type of manuscript: Protocol

For peer review only

ABSTRACT

Introduction:

Amblyopia is an important public health concern associated with functional vision loss and detrimental impact on the physical and mental well-being of children. The gold standard for diagnosis of amblyogenic conditions involves screening by ophthalmologists and orthoptists. The bloom of technology enables the use of home-based screening tools to detect these conditions at an early stage by the layperson in community, which could reduce the burden of screening in the community, especially during restrictions associated with the COVID-19 pandemic. Here, we propose a systematic review aiming to evaluate the accuracy and reliability of home-based screening tools compared to the existing gold standard.

Methods & Analysis:

We aim to search for studies involving home-based screening tools for amblyopia among children aged under 18 years. Oxford Centre for Evidence-Based Medicine Level 4 evidence and above will be included, without language or time restrictions. The following platforms will be searched from inception to 31th August 2021: Pubmed, Medline, The Cochrane Library, Embase, Web of Science Core Collection and Clinicaltrials.gov. Two independent reviewers will identify studies for inclusion based on a screening questionnaire. The search and screening will start on 14th August 2021 until 1st October 2021.We aim to complete our data analysis by 30th November 2021. Risk of bias will be assessed using the QUADAS-2 tool for diagnostic accuracy studies only. Our primary outcome measure is the diagnostic accuracy of home-based screening tools, whilst secondary outcome measures include validity, feasibility, reproducibility, and cost effectiveness, where available.

Ethics & Dissemination:

Ethical approval is not necessary as no primary data will be collected. The findings will be disseminated through presentations at scientific meetings and peer-reviewed journal publication.

Prospero registration number: CRD42021233511

Strengths and limitations of this study:

For peer review only - http://bmjopen.bmj.com/site/about/guidelines.xhtml

- This will be the first systematic review evaluating the accuracy and reliability of home-based screening tools for amblyopia.
- Published and unpublished literature without language or time restrictions will be included.
- Protocol methodology are based on principles extracted from the Cochrane Collaboration.
- The main limitation could be a scarcity of randomised controlled trials and diagnostic accuracy studies involving home-based screening tools.
- The broad search strategy should help ensure that all relevant literature is included.

For peer review only

For peer review only - http://bmjopen.bmj.com/site/about/guidelines.xhtml

INTRODUCTION

Amblyopia is one of the commonest preventable causes of vision loss affecting children. It continues to represent a significant public health concern, affecting 2-5% of the population.[1,2,3] Amblyopia is usually associated with visual deprivation early in life,[4] due to amblyogenic risk factors which include uncorrected refractive errors, astigmatism, congenital pathologies or media opacities that causes stimulus deprivation, and abnormal binocular interaction from strabismus.[5,6,7] Children with amblyopia are characterised by monocular or binocular visual deficits, including reduced visual acuity, contrast sensitivity, contour integration and depth perception without observable ocular pathologic features.[8]

Amblyopia is largely asymptomatic initially, but untreated amblyopia resulting in vision loss can lead to problems at school, bullying, reduced quality of life, lifelong consequences on future occupation choices and mental health issues.[9,10] Contrary to the traditional notion that amblyopia treatment may be ineffective for children above 7 years old,[11] the Paediatric Eye Disease Investigator Group (PEDIG) studies showed that treatment of amblyopia may still be effective in children aged 7 to 17 years,[12,13] with the effectiveness of treatment becoming significantly reduced with time.[14] Whilst amblyopia is treatable, the key to manage this disorder effectively is early detection by screening. Screening for amblyopia have been introduced in the 1950s and advocated in many countries.[15] Many screening programmes have been unsuccessful, with an estimation of less than 25% of preschool-aged children being screened through a government or private program in the United States.[16] In addition, up to 60% of primary care providers do not perform vision screening on preschool-aged children, and others perform screening inconsistently.[16] Significant barriers to traditional vision screening include cost, limited access to healthcare and a limited number of qualified screeners available.[17] Hence, a variety of methodologies for vision screening have been trialled, including the use of home-based amblyopia screening tools, to help overcome these barriers to vision screening.[18]

The coronavirus disease 2019 (COVID-19) pandemic illustrates the increasingly important role of telemedicine as a method of clinician-patient interaction. The use of home-based screening tools for amblyopia are increasingly advocated as social distancing is practised to minimise the risk of viral transmission.[19,20] Furthermore, COVID-19 related restrictions and lockdowns may have resulted in many children missing out opportunities for amblyopia screening.[19] Home-based screening tools may offer a solution,[21] but its role has not been rigorously assessed and evaluated by systematic review. Here, we propose a systematic review to evaluate home-based amblyopia screening tools.

For peer review only - http://bmjopen.bmj.com/site/about/guidelines.xhtml

METHODS AND ANALYSIS

This protocol is drafted according to the Preferred Reporting Items for Systematic Review and Meta-Analysis Protocols (PRISMA-P) checklist.[22]

Eligibility criteria for studies

The eligible study characteristics for this systematic review are defined according to the Population, Intervention, Comparison, Outcome and Study Design (PICOS)[23]study strategy outlined in Table 1.

Table 1 Eligibility criteria

PICOS strategy	Inclusion criteria	Exclusion criteria
**Population**	Studies involving screening for amblyopia in children aged under 18 years old.	Studies involving adults aged 18 years old and above
**Intervention**	Home-based screening tools including:<br>i) Internet or web-based visual acuity screening tools;<br>ii) Mobile applications used to screen for conditions contributing to amblyopia;<br>iii) Novel home-based gadgets or instruments used to screen for conditions contributing to amblyopia.	Orthoptist-led or ophthalmologist-led amblyopia screening tests including:<br>i) Standard logMAR (or equivalent) visual acuity measurement charts<br>ii) Comprehensive eye examination using slit lamp or ocular motility examination<br>iii)Autorefractors or photoscreeners
**Comparison/Control**	Orthoptist-led or ophthalmologist-led amblyopia screening	Not applicable

For peer review only - http://bmjopen.bmj.com/site/about/guidelines.xhtml

**Outcomes**	Primary outcome measure: Diagnostic accuracy of home-based amblyopia screening tools<br><br>Secondary outcome measures, where available: validity, feasibility, reproducibility, cost effectiveness.	i) Studies not reporting outcomes related to amblyopia screening;<br>ii) Epidemiological studies reporting prevalence of amblyopia.
**Study Design**	According to the Oxford Centre for Evidence-Based Medicine (CEBM) Level 4 evidence and above will be included[24]	CEBM Level 5 evidence and below will be excluded.

Information sources

The following electronic searches will be included in this systematic review

I. Ovid MEDLINE® 1946 to present
II. Pubmed
III. The Cochrane Library
IV. Embase 1974 to present
V. Web of Science Core Collection (1970 to present).
VI. Clinicaltrials.gov

Sources I and IVwill be searched through the Ovid platform separately.

Other sources

Publications of all formats, including protocols and conference abstracts, not limited by year and language will be included.

To ensure literature saturation, references of included studies will be searched and included if meeting inclusion criteria. Authors of studies with insufficient data published will be contacted via email in attempt to extract relevant outcome data. If there is no response from these authors

For peer review only - http://bmjopen.bmj.com/site/about/guidelines.xhtml

after 14 days, another email will be sent to attempt to establish contact. If there is still no response after 14 days, these studies will be excluded.

Search strategy

The search strategy was developed after convening with a research services consultant with experience in systematic review. The search terms 'amblyopia', 'visual acuity', 'vision screening', 'home', 'web', 'internet' 'app', 'smartphone', and 'mobile' were entered into the electronic search platforms. A sample of the full search strategy using the electronic databases listed is available in the online supplementary Appendix 1.

Study records

Data Management

EndNote V.X9 (Thomson Reuters, New York, New York, USA) reference management software will be used for data management.

Selection of studies

Two independent screeners (SS and CSC) shall follow a three-stage screening method, according to a screening questionnaire (online supplementary Appendix 2). After the screening of titles and abstracts, SS and CSC will compare and attempt to resolve any disagreements on the inclusion of articles, where applicable. If any disagreement remains, opinion will be sought from the third arbitrator (HJK). We aim to start the search by 14th August 2021 and complete the screening process by 1st October 2021.

Data collection

Our data collection tool adapted from the Cochrane Collaboration is included in online supplementary Appendix 3. A preliminary data collection form was first drawn and piloted among the authors of this study before use. The following data shall be collected: study design, number of included patients, duration of study, method of intervention used, index test and reference test where applicable. Outcomes pertinent to the quality of diagnostic studies

For peer review only - http://bmjopen.bmj.com/site/about/guidelines.xhtml

including investigators conducting test, subjects receiving test, method of interpretation of test, blinding of participants or investigators and withdrawal rate will also be included.

Outcome Measures and Prioritisation

Our primary outcome measure of interest will be the diagnostic accuracy of home-based screening tools in detecting amblyopia compared to the existing gold standard which is diagnosis made by orthoptists or ophthalmologists. Outcomes from diagnostic accuracy studies such as sensitivity, specificity, positive predictive value (PPV), and negative predictive value (NPV) will be prioritized as the primary outcome as they will translate into meaningful endpoints for comparing the effectiveness of home-based screening tools against the gold standard. The secondary outcome measures, or surrogate measures of this review where available may include validity, feasibility, reproducibility, and cost effectiveness of these home-based screening tools compared to existing gold standard screening. These will be reported in appropriate statistical measures if represented by studies with large enough sample sizes. As some outcomes may be reported as a composite measure, we will extract all composite and individual outcomes as reported in the studies.

Risk of bias assessment

Risk of bias assessment will be done for diagnostic accuracy studiesonly.The quality of diagnostic accuracy studies will be assessed using the QUADAS-2 tool(online supplementary Appendix 4).[25]These judgments will be made independently by two review authors (SS, CSC) and any disagreements discussed with the third arbitrator (HJK). If our risk of bias assessment shows lack of good quality studies with adequate sample sizes, statistical measures will not be summarized quantitatively and vice versa.

Data analysis

Scoping searches suggest that mainly observational studies will be returned by our search strategy with few relevant randomised controlled trials (RCTs)Weighted means for primary outcome measures (such as sensitivity, specificity, PPV, NPV) will only be calculated if multiple RCTs or good quality large scale prospective studies are identified. Otherwise, we

For peer review only - http://bmjopen.bmj.com/site/about/guidelines.xhtml

shall perform a qualitative review summarising the available evidence of good quality studies in the form of tables to explain the characteristics of and results of the included studies as well as relevant p-values. This will be followed by a narrative synthesis of secondary outcome measures such as validity, feasibility, reproducibility, or cost effectiveness of home-based screening tools. We aim to complete our data analysis by 30th November 2021.

Confidence in cumulative estimate

The quality of evidence for all outcomes will be judged using the Grading of Recommendations Assessment, Development and Evaluation working group methodology (GRADE)[26] and will be judged as high (further research is very unlikely to change our confidence in the estimate of effect), moderate (further research is likely to have an important impact on our confidence in the estimate of effect and may change the estimate), low (further research is very likely to have an important impact on our confidence in the estimate of effect and is likely to change the estimate), or very low (very uncertain about the estimate of effect).

Patient and Public Involvement statement

As this systematic review does not involve recruitment of patients for research, patient and public involvement is not applicable.

Ethics and dissemination

As this systematic review does not involve recruiting patients, independent ethical approval is not required. The findings of this systematic review shall be disseminated through presentations at scientific meetings, as well as peer-reviewed journal publication. Any data generated from this systematic review will be made available from the corresponding author on reasonable request.

Discussion

To our knowledge, this is the first systematic review aiming to compare home-based screening tools and existing screening services offered through ophthalmologists and orthoptists to diagnose amblyopia. We adhered to the PRISMA-P checklist in drafting this protocol. Through publication of this protocol, we aim to provide transparency in the methodology of our systematic review. This should increase internal validity by preventing publication bias and should help avoid study duplication.

For peer review only - http://bmjopen.bmj.com/site/about/guidelines.xhtml

Author's contributions:

SS: Concept, methodology, drafting of protocol, piloting of questionnaires, revision and final approval of manuscript

CC: Drafting of protocol (introduction) and critically reviewing manuscript

HK: Critically reviewing manuscript

MGT: Supervision, critically reviewing protocol and manuscript

SRR: Supervision, concept, methodology, peer-review of search strategy, questionnaires, critically reviewing protocol and manuscript

Funding statement:

This work is funded by the National Institute for Health Research (NIHR).SRR is funded by a National Institute for Health Research (NIHR) Doctoral Fellowship Award ID: NIHR300155. MGT is also supported by the NIHR (CL-2017-11-003) and the Ulverscroft foundation. This paper presents independent research funded by the NIHR. The views expressed are those of the author(s) and not necessarily those of, the Ulverscroft Foundation, the NHS, the NIHR or the Department of Health and Social Care.

Competing interest: None declared

For peer review only - http://bmjopen.bmj.com/site/about/guidelines.xhtml

References

1. Powell C, Hatt SR. Vision screening for amblyopia in childhood. *Cochrane Database Syst Rev* 2009; 8:(3). https://www.cochranelibrary.com/cdsr/doi/10.1002/14651858.CD005020.pub3/full (accessed on 22 Feb 2021)
2. Friedman DA, Repka MX, Katz J, et al: Prevalence of amblyopia and strabismus in White and African-American children aged 6 through 71 months: The Baltimore Pediatric Eye Disease Study. *Ophthalmology* 2009; 116: 2128-2134
3. Multi-ethnic Pediatric Eye Disease Study Group. Prevalence of amblyopia and strabismus in African American and Hispanic children ages 6 to 72 months the multi-ethnic pediatric eye disease study. *Ophthalmology* 2008; 115: 1229-1236
4. Levi DM. Progress and paradigm shifts in spatial vision over the 20 years of ECVP. *Perception* 1999;28(12):1443-59.
5. Rahi_J, Logan_S, Timms_C et al. Risk, causes, and outcomes of visual impairment after loss of vision in the non-amblyopic eye: a population-based study. *Lancet* 2002; 360 (9333):597-602.
6. Repka M, Simons K, and Kraker R. Laterality of amblyopia. *Am J Ophthalmol* 2010; 150: pp. 270-274
7. Shaw DE, Fielder AR, Minshull C et al. Amblyopia – Factors influencing age of presentation. *Lancet* 1988; 2: 207-209
8. Lan W, Zhao F, Li Z et al. Validation and cost-effectiveness of a home-based screening system for amblyopia. *Ophthalmology* 2012 ;119(6):1265-71.
9. Jonas DE, Amick HR, Wallace IF et al. Vision screening in children aged 6 months to 5 years: evidence report and systematic review for the US Preventive Services Task Force. *JAMA* 2017; 318(9):845-58.
10. Tandon AK, Velez FG, Isenberg SJ, et al: Binocular inhibition in strabismic patients is associated with diminished quality of life. *J AAPOS* 2014; 18:423-426
11. Assaf AA. The sensitive period: transfer of fixation after occlusion for strabismic amblyopia. *Br J Ophthalmol* 1982; 66: 64–70.
12. Scheiman MM, Hertle RW, Beck RW et al. Randomized trial of treatment of amblyopia in children aged 7 to 17 years. *Arch Ophthalmol* 2005 Apr 1;123(4):437-47.
13. Scheiman MM, Hertle RW, Kraker RT, et al. Patching vs atropine to treat amblyopia in children aged 7 to 12 years: a randomized trial. *Arch Ophthalmol* 2008;126(12):1634-1642.

For peer review only - http://bmjopen.bmj.com/site/about/guidelines.xhtml

14. Holmes JM, Lazar EL, Melia BM, et al. Effect of age on response to amblyopia treatment in children. *Arch Ophthalmol* 2011;129(11):1451-57
15. Gundersen T. Glaucoma and amblyopia ex anopsia, two preventable forms of blindness. *J Am Med Assoc* 1954; 156: 933–5.
16. Hered RW, Murphy S, Clancy M. Comparison of the HOTV and Lea symbols charts for preschool vision screening. *J Pediatr Ophthalmol Strabismus* 1997; 34: 24-8.
17. Longmuir SQ, Pfeifer W, Shah SS et al. Validity of a layperson-administered Web-based vision screening test. *J AAPOS* 2015 ; 19(1): 29-32.
18. Tamez-Tamez VE, Ruiz-Lozano RE. Evaluating amblyopia during the era of COVID-19. *Graefes Arch Clin Exp Ophthalmol*. 2020;258(12):2857-2859.
19. Painter, S, Ramm, L, Wadlow, L. et al Parental Home Vision Testing of Children During Covid-19 Pandemic. *British and Irish Orthoptic Journal*, 17(1), 13–19
20. Samanta A, Mauntana S, Barsi Z, Yarlagadda B, Nelson PC. Is your vision blurry? A systematic review of home-based visual acuity for telemedicine. *J Telemed Telecare* 2020 Jan 1:1357633X20970398.
21. Kawamoto, K, Stanojcic, N, Li, JO et al. Visual Acuity Apps for Rapid Integration in Teleconsultation Services in all Resource Settings, *Asia Pac J Ophthalmol*:2021 https://journals.lww.com/apjoo/pages/articleviewer.aspx?year=9000&issue=00000&article=99702&type=Abstract (accessed on 29 Feb 2021)
22. Shamseer L, Moher D, Clarke M, *et al*. Preferred reporting items for systematic review and meta-analysis protocols (PRISMA-P) 2015: elaboration and explanation. *BMJ* 2015; 349: g7647.
23. Higgins JP, Green S. Defining the review question and developing criteria for including studies, in Cochrane handbook for systematic reviews of interventions. *Wiley Online Library*, 2008: 1–83.
24. CEBM. Oxford Centre for Evidence-based Medicine – Levels of Evidence (March 2009). taken from https://www. cebm. net/ 2009/ 06/oxford- centre- evidence- based-medicine- levels- evidence- march-2009/ (accessed 29 Feb 2021)
25. Whiting P, Rutjes A, Westwood M, et al. QUADAS-2: an updated quality assessment tool for diagnostic accuracy studies. In: *Abstracts of the 19th Cochrane Colloquium*; 2011 19-22 Oct. https://abstracts.cochrane.org/2011-madrid/quadas-2-updated-quality-assessment-tool-diagnostic-accuracy-studies (accessed on 27 Feb 2011)

For peer review only - http://bmjopen.bmj.com/site/about/guidelines.xhtml

26. Iorio A, Spencer FA, Falavigna M, *et al*. Use of grade for assessment of evidence about prognosis: rating confidence in estimates of event rates in broad categories of patients. *BMJ* 2015; 350: h870.

Acknowledgement:

We thank Selina Lock, Research Services Consultant at the University of Leicester David Wilson Library, for providing expert guidance in our systematic search strategy.

For peer review only - http://bmjopen.bmj.com/site/about/guidelines.xhtml

Appendices

Appendix 1: Sample search strategy – MEDLINE

1. exp Amblyopia/ or amblyop*.mp. or amblyog*.mp.

2. ((assess* or diagnos* or detect* or screen* or test*) adj4 (vision* or visual*)).mp.

3. exp visual acuity/

4. exp vision screening/ or screen*.mp.

5. ((assess* or diagnos* or detect*or screen* or test*) adj4 (communit* or population*)).mp.

6. ((assess* or diagnos* or detect* or screen* or test*) adj4 program*).mp.

7. 2 or 3 or 4 or 5 or 6

8. (home* or internet* or web* or app* or computer* or smartphone* or mobile*).mp. or Mobile Applications/ or Smartphone Applications/

9. 1 and 7 and 8

10. limit 9 to "all child (0 to 18 years)"

For peer review only

For peer review only - http://bmjopen.bmj.com/site/about/guidelines.xhtml

Appendix 2: Screening questionnaire

Instructions for screeners: Tick the appropriate box per screening question. If "no" at any stage, exclude. If "yes" or "unclear", proceed to next stage; if "yes" at Stage 3, include. If "unclear" at Stage 3, contact study authors for further information and/or seek verdict from third arbitrator.

Stage 1: Title Screening

1a) Does the study represent Level IV evidence or above, i.e. case series, cohort studies, case-control studies, randomised controlled trials (RCTs) and systematic reviews?

Yes	
No	
Unclear	

1b) Does the study involve home-based screening methods for amblyopia in children under 18 years of age?

Yes	
No	
Unclear	

Stage 2: Abstract Screening

2a) Does the study represent Level IV evidence or above, i.e. case series, cohort studies, case-control studies, randomised controlled trials (RCTs) and systematic reviews?

Yes	
No	
Unclear	

2b) Does the study involve home-based screening methods for amblyopia in children under 18 years of age?

Yes	
No	
Unclear	

Stage 3: Full text Screening

3a) Does the study represent Level IV evidence or above, i.e. case series, cohort studies, case-control studies, randomised controlled trials (RCTs) and systematic reviews?

Yes	
No	
Unclear	

3b) Does the study involve home-based screening methods for amblyopia in children under 18 years of age?

Yes	
No	
Unclear	

For peer review only

For peer review only - http://bmjopen.bmj.com/site/about/guidelines.xhtml

Appendix 3: Data extraction tool, adapted from the Cochrane Collaboration

Methods

Aim of study	
Study design	
Inclusion criteria	
Exclusion criteria	
Methods of recruitment	
Methods of randomisation (if applicable)	
Number of patients	

Specific to diagnostic accuracy studies

Personnel conducting index test	
Personnel conducting gold standard test	
Subjects receiving test	
Blinding (if applicable)	
Index test	
Reference test	
Personnel interpreting test results	
Withdrawal rate/loss to follow up<br>If not specified state so	
Sensitivity(including CI)	
Specificity(including CI)	
False positive	
False negative	
Correlation coefficient including p-values	

Specific to other evaluation studies

Personnel conducting index test	
Personnel conducting reference test	
Subjects receiving test	
Blinding (if applicable)	
Index test	
Reference test	
Personnel interpreting test results	
Withdrawal rate/loss to follow up	
Results reported (appropriate statistical measures)	
Economic consideration/cost required	

For peer review only - http://bmjopen.bmj.com/site/about/guidelines.xhtml

Appendix 4: QUADAS-2 tool

Domain 1: Patient selection

A. Risk of bias<br>Describe methods of patient selection:	Low	High	Unclear
Was a consecutive or random sample of patients enrolled?			
Was a case-control design avoided?			
Did the study avoid inappropriate exclusions?			
Could the selection of patients have introduced bias?			
B. Concerns regarding applicability	Low	High	Unclear
Is there concern that the included patients do not represent the actual spectrum of patients in practice?			

Domain 2: Index test(s)

Risk of bias<br>Describe the index text and how is it conducted and interpreted:	Low	High	Unclear
Were the index test results interpreted without knowledge of the results of the reference standard?			
If a threshold was used, was it pre-specified?			
Was the index test described in sufficient detail to enable replication of the test?			
Could the index test, its conduct, or its interpretation have introduced bias?			
B. Concerns regarding applicability	Low	High	Unclear
Is there concern that the index test, its conduct, or interpretation differ from the review question?			

Domain 3: Reference standard

For peer review only

For peer review only - http://bmjopen.bmj.com/site/about/guidelines.xhtml

Risk of bias<br>Describe the index text and how is it conducted and interpreted:	Low	High	Unclear
Is the reference standard likely to correctly classify the target condition?			
Were the reference standard results interpreted without knowledge of the results of the index test?			
Was the standard test described in sufficient detail to enable replication of the test?			
Could the reference standard, its conduct, or its interpretation have introduced bias?			
B. Concerns regarding applicability	Low	High	Unclear
Is there concern that the target condition as defined by the reference standard does not match the review question?			

Domain 4: Flow and timing

Risk of bias	Low	High	Unclear
Was there an appropriate interval between index test(s) and reference standard, so that the target condition did not change between two tests?			
Did all patients receive a reference standard?			
Did all patients receive the same reference standard?			
Were all patients included in the analysis?			
Could the patient flow have introduced bias?			

For peer review only

For peer review only

For peer review only - http://bmjopen.bmj.com/site/about/guidelines.xhtml

Reporting checklist for protocol of a systematic review and meta analysis.

Based on the PRISMA-P guidelines.

Instructions to authors

Complete this checklist by entering the page numbers from your manuscript where readers will find each of the items listed below.

Your article may not currently address all the items on the checklist. Please modify your text to include the missing information. If you are certain that an item does not apply, please write "n/a" and provide a short explanation.

Upload your completed checklist as an extra file when you submit to a journal.

In your methods section, say that you used the PRISMA-Preporting guidelines, and cite them as:

Moher D, Shamseer L, Clarke M, Ghersi D, Liberati A, Petticrew M, Shekelle P, Stewart LA. Preferred Reporting Items for Systematic Review and Meta-Analysis Protocols (PRISMA-P) 2015 statement. Syst Rev. 2015;4(1):1.

For peer review only

		Reporting Item	Page Number
**Title**			
Identification	#1a	Identify the report as a protocol of a systematic review	1
Update	#1b	If the protocol is for an update of a previous systematic review, identify as such	NA

**Registration**			
	#2	If registered, provide the name of the registry (such as PROSPERO) and registration number	2
**Authors**			
Contact	#3a	Provide name, institutional affiliation, e-mail address of all protocol authors; provide physical mailing address of corresponding author	1
Contribution	#3b	Describe contributions of protocol authors and identify the guarantor of the review	10
**Amendments**			
	#4	If the protocol represents an amendment of a previously completed or published protocol, identify as such and list changes; otherwise, state plan for documenting important protocol amendments	NA
**Support**			
Sources	#5a	Indicate sources of financial or other support for the review	10
Sponsor	#5b	Provide name for the review funder and / or sponsor	10
Role of sponsor or funder	#5c	Describe roles of funder(s), sponsor(s), and / or institution(s), if any, in developing the protocol	10
**Introduction**			
Rationale	#6	Describe the rationale for the review in the context of what is	4

For peer review only

		already known	
Objectives	#7	Provide an explicit statement of the question(s) the review will address with reference to participants, interventions, comparators, and outcomes (PICO)	5-6
**Methods**			
Eligibility criteria	#8	Specify the study characteristics (such as PICO, study design, setting, time frame) and report characteristics (such as years considered, language, publication status) to be used as criteria for eligibility for the review	5-6
Information sources	#9	Describe all intended information sources (such as electronic databases, contact with study authors, trial registers or other grey literature sources) with planned dates of coverage	6
Search strategy	#10	Present draft of search strategy to be used for at least one electronic database, including planned limits, such that it could be repeated	7
Study records - data management	#11a	Describe the mechanism(s) that will be used to manage records and data throughout the review	7
Study records - selection process	#11b	State the process that will be used for selecting studies (such as two independent reviewers) through each phase of the review (that is, screening, eligibility and inclusion in meta-analysis)	7
Study records - data collection	#11c	Describe planned method of extracting data from reports (such as piloting forms, done independently, in duplicate), any	7

For peer review only

process		processes for obtaining and confirming data from investigators	
Data items	#12	List and define all variables for which data will be sought (such as PICO items, funding sources), any pre-planned data assumptions and simplifications	7
Outcomes and prioritization	#13	List and define all outcomes for which data will be sought, including prioritization of main and additional outcomes, with rationale	8
Risk of bias in individual studies	#14	Describe anticipated methods for assessing risk of bias of individual studies, including whether this will be done at the outcome or study level, or both; state how this information will be used in data synthesis	8
Data synthesis	#15a	Describe criteria under which study data will be quantitatively synthesised	8
Data synthesis	#15b	If data are appropriate for quantitative synthesis, describe planned summary measures, methods of handling data and methods of combining data from studies, including any planned exploration of consistency (such as I2, Kendall's τ)	8
Data synthesis	#15c	Describe any proposed additional analyses (such as sensitivity or subgroup analyses, meta-regression)	8
Data synthesis	#15d	If quantitative synthesis is not appropriate, describe the type of summary planned	8
Meta-bias(es)	#16	Specify any planned assessment of meta-bias(es) (such as publication bias across studies, selective reporting within	NA

For peer review only

		studies)	
Confidence in cumulative evidence	#17	Describe how the strength of the body of evidence will be assessed (such as GRADE)	9

The PRISMA-P elaboration and explanation paper is distributed under the terms of the Creative Commons Attribution License CC-BY. This checklist was completed on 27. March 2021 using https://www.goodreports.org/, a tool made by the EQUATOR Network in collaboration with Penelope.ai

For peer review only
